# Association between alcohol and urolithiasis: a mendelian randomization study

**DOI:** 10.1007/s00240-023-01472-0

**Published:** 2023-08-15

**Authors:** Shijian Yang, Wenyue Tan, Baian Wei, Chiming Gu, Siyi Li, Shusheng Wang

**Affiliations:** 1 Guangdong Provincial Hospital of Chinese Medicine, Guangzhou, China; 2 The Second Clinical College, Guangzhou University of Chinese Medicine, Guangzhou, China

**Keywords:** Causality, Alcohol, Mendelian randomization, Urolithiasis, FinnGen database

## Abstract

The causal relationship between alcohol and urolithiasis remains uncertain, despite previous observational studies reporting an association between the two. To determine the causality, we conducted a two-sample Mendelian randomization (MR) analysis. In this study, we aimed to investigate the causal relationship between alcohol and kidney stones using a two-sample MR approach. Two sets of genetic instruments were utilized in the analysis, both of which were derived from publicly available genetic summary data. The first set consisted of 73 single-nucleotide polymorphisms (SNPs) robustly linked to alcohol intake frequency (AIF) and the second set was comprised of 69 SNPs associated with alcohol consumption (AC). Our MR analysis was performed using several methods including the inverse-variance weighted (IVW) method, weighted median method, MR-Egger regression, MR Pleiotropy RESidual Sum and Outlier test. Our results from the MR analysis revealed a borderline significant association between AIF and the risk of urolithiasis. This was established through the use of the IVW method (OR (95% CI) = 1.29 (1.02, 1.65), *p* = 0.036) and the weighted median approach (OR (95% CI) = 1.44 (1.10, 1.89), *p* = 0.008). The MR-Egger model also yielded similar risk estimates (OR (95% CI) = 1.39 (0.66, 2.93), *p* = 0.386), although the relationship was not statistically significant. Sixty-eight SNPs were identified as having a substantial and independent link with AC. However, the IVW approach revealed no significant effect of AC on the risk of urolithiasis (OR (95% CI) = 0.74 (0.48, 1.14), *p* = 0.173). The MR analysis suggested a potential causal association between alcohol intake frequency and the risk of urolithiasis, but not alcohol consumption.

**Supplementary Information:**

The online version contains supplementary material available at 10.1007/s00240-023-01472-0.

## Introduction

Urolithiasis is a growing public health issue with significant healthcare costs and morbidity [1, 2]. The excessive consumption of alcohol has been well-established as a major contributor to both mortality and disability. [3] However, the interplay between moderate alcohol intake and the urolithiasis risk remains multifaceted and merits further investigation.

Alcoholic beverages are a complex mixture of chemicals, and their consumption has been linked to a range of health outcomes. The primary constituent of alcoholic beverages is ethanol, whose metabolism produces acetaldehyde, capable of causing DNA damage, hindering DNA synthesis and repair, and inducing inflammation and oxidative stress, leading to lipid peroxidation [4]. Given the widespread nature of alcohol consumption, it is imperative to unravel the risks and benefits associated with it on a population level.

Several prospective studies [5–7] indicate a potential inverse association between alcohol and urolithiasis, while a meta-analysis has reported a dose-dependent correlation between alcohol consumption and urolithiasis incidence [8]. However, conflicting evidence exists, as some studies have failed to demonstrate a protective effect of alcohol consumption against urolithiasis [9–11]. Observational studies are prone to residual confounding, which is important because urolithiasis is linked to other factors such as obesity, diabetes, hypertension, and metabolic syndrome [12–14]. As a result, the causality of the links between alcohol and urolithiasis remains unknown.

In this study, Mendelian randomization (MR) is employed to strengthen causal inference by using genetic variations as instrumental factors for exposure (e.g., alcohol consumption) [15]. MR leverages genetic variations that influence modifiable risk factors to estimate a causal association between exposure and outcome. Genetic variations are randomly assigned during meiosis, independent of confounders, and are not affected by outcomes, making MR less susceptible to confounding and reverse causation compared to traditional observational methods [16]. In recent years, there has been a surge of interest in applying MR to public health policies and clinical decision-making. This work aims to determine the impact of alcohol on urolithiasis risk.

## Materials and method study overview

**Fig. 1:**
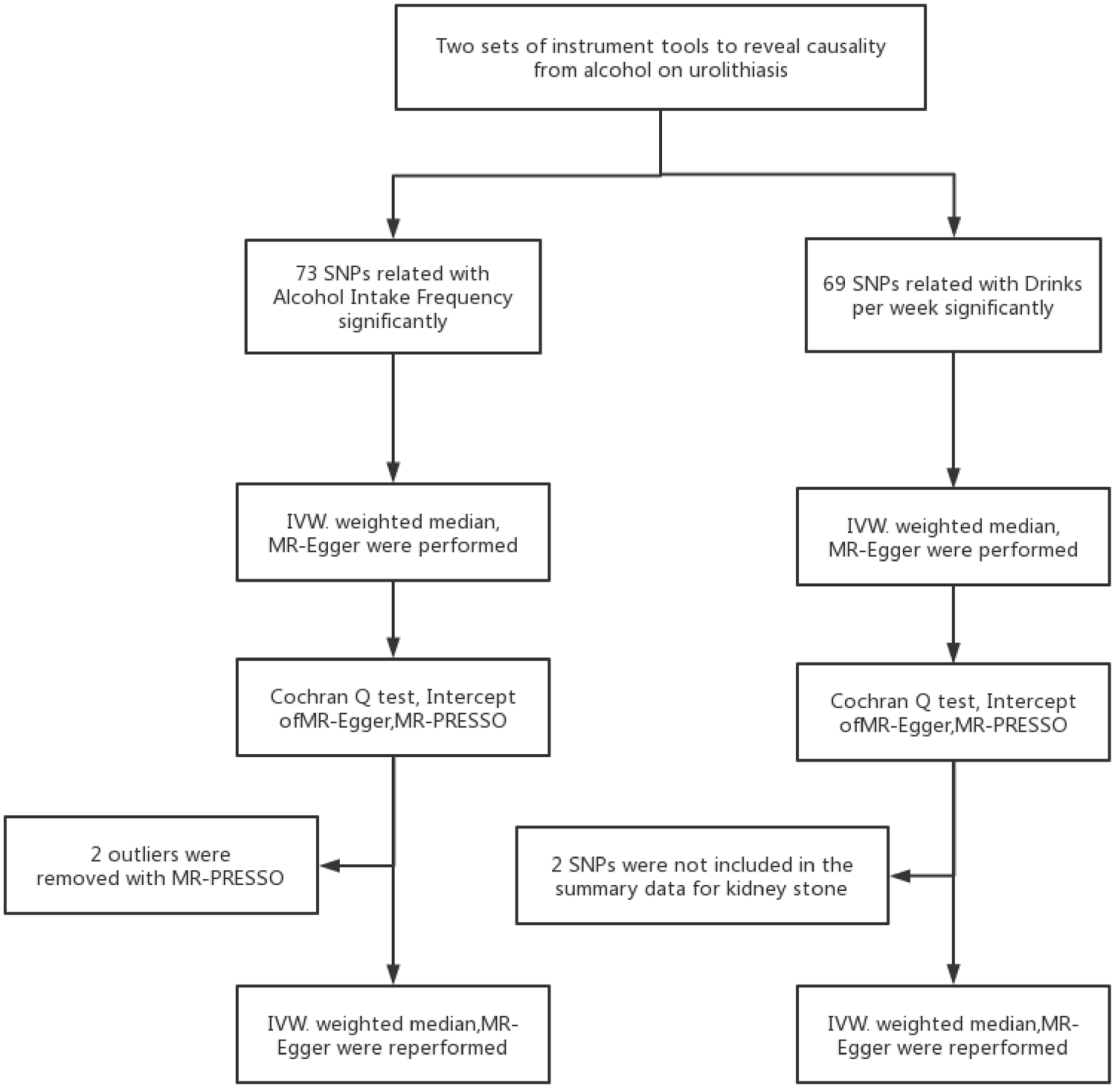
Workflow of Mendelian randomization study revealing causality from alcohol on urolithiasis. *IVW* inverse variance weighted; *MR* medilian randomization; MR-PRESSO; MR pleiotropy RESidual Sum and outlier; SNP single-nucleotide polymorphisms

The conceptual framework of the two-sample MR analysis is illustrated in Fig. 1. In this study, the use of single nucleotide polymorphisms (SNPs) as instrumental variables (IVs) aims to investigate the causal relationship between alcohol consumption and urolithiasis risk. Three assumptions must be satisfied for this approach: (1) the SNPs must have a strong association with alcohol consumption; (2) the SNPs should not affect confounders that may impact the relationship between exposure and outcome; and (3) the SNPs should only impact the outcome through the exposure, and not through any other pathways.

### Genetic variants associated with alcohol intake frequency and alcohol consumption

The study utilized two sets of genetic instruments to assess the causal relationship between alcohol consumption and urolithiasis. The primary genetic instruments were obtained from the MR-base database [17]. Alcohol intake frequency (AIF) was recorded as an ordinal categorical response, which consisted of “never,” “only on special occasions,” “once to three times a month,” “once or twice a week,” “three or four times a week,” and “daily or almost daily”. 99 SNPs were identified as being significantly associated with AIF (*p* < 5 × 10^–8^, linkage disequilibrium [LD] ${r}^{2}$ < 0.01). The details of these 99 SNPs can be found in Table S1. Of the 80 SNPs, the F-statistics of more than the conventional value of 10 indicate a strong potential for these instruments to predict AIF.

To establish a clearer understanding of the causal relationship between alcohol consumption and urolithiasis, we have generated a new set of genetic instruments that are based on the weekly alcohol consumption (DPW) of individuals. This data was collected from a recent genome-wide association study (GWAS) that was conducted by the Data Repository for the University of Minnesota and includes data from 60 different GWAS studies that were conducted on a total of up to 3.4 million participants from four major ancestry groups and we used the data derived from the European ancestry subgroup. [18] The study focuses on nicotine and substance use, providing a comprehensive understanding of the influence of alcohol consumption on the development of urolithiasis. Out of the 76 SNPs found to be significantly associated with alcohol consumption (*p* < 5 × 10^−8^, linkage disequilibrium [LD] ${r}^{2}$ < 0.01), information on the 76 SNPs can be found in Table S2. 71 SNPs had F-statistics larger than the conventional value of 10, suggesting that these instruments possess a strong potential in predicting alcohol consumption.

### GWAS summary data for urolithiasis

The FINNGEN database was employed to gather the GWAS summary data for the investigation of urolithiasis [19]. Regarding the database, the diagnosis of urolithiasis is based on clinician diagnosis and classified according to ICD-10 criteria. The dataset consisted of 7433 urolithiasis cases and 301,094 control cases. Summary data was sourced from FINNGEN, but two SNPs were excluded due to their absence in the summary data for kidney stones. Following harmonization, four SNPs (s1104608, rs117799466, rs1894544, and rs62097995) were removed as they were palindromic and had intermediate allele frequencies. The SNP rs2159935 was also eliminated due to incompatible alleles. In conclusion, 73 SNPs were selected for further analysis of AIF. Additionally, 69 SNPs were found to be related to DPW, with SNP rs1714507 being excluded as it was palindromic with intermediate allele frequency.

### Statistical analyses

We conducted a harmonized analysis of the genetic impact of alcohol on urolithiasis using multiple MR techniques. This was done to account for the potential presence of horizontal pleiotropy, which could affect the validity of our results. The primary MR analysis employed the inverse variance weighted (IVW) method, which assumes that the genetic instruments affect the outcome solely through the exposure of interest. To enhance the robustness of our findings, we also employed the MR-Egger and weighted median methods, which have been demonstrated to be effective in a broader range of scenarios, albeit with lower efficiency and wider confidence intervals [20]. These approaches aimed to supplement the results obtained from the IVW method and provide a more comprehensive understanding of the causality of alcohol on urolithiasis [21].

Sensitivity analysis plays a crucial role in MR investigations for uncovering the presence of pleiotropy and avoiding violation of heterogeneity in MR estimates. In our study, we utilized the Cochran *Q*-derived *p*-value of less than 0.05 from the IVW method as a marker for potential horizontal pleiotropy. Additionally, the MR-Egger regression intercept was employed as an indicator of directional pleiotropy, where a *p*-value of less than 0.05 was considered to indicate the presence of directional pleiotropy [22]. The MR-PRESSO (MR-Pleiotropy Residual Sum and Outlier) method was employed to investigate and rectify horizontal pleiotropy [21]. Additionally, the MR-PRESSO method was applied to rectify and analyze horizontal pleiotropy. It consists of three steps: (1) identifying horizontal pleiotropy, (2) correcting for horizontal pleiotropy through the removal of outliers, and (3) testing for significant differences in the causal estimates before and after the removal of outliers. The MR-PRESSO approach is considered to be less biased and more accurate than the IVW and MR-Egger methods when the percentage of horizontal pleiotropy variations is less than 10% [23]. Additionally, a leave-one-out analysis was performed to investigate whether a single SNP had a significant impact on the MR estimate. To further evaluate the potential confounding effect of pleiotropy, we utilized the PhenoScanner tool (http://www.phenoscanner.medschl.cam.ac.uk/) during our analysis. To test the credibility of our study, we also carried out the power test. The Two-Sample MR package (version 0.5.6) was used to conduct all the analyses, in combination with R version 4.2.1.

## Result

### Causal effect of alcohol intake frequency on urolithiasis

**Fig. 2:**
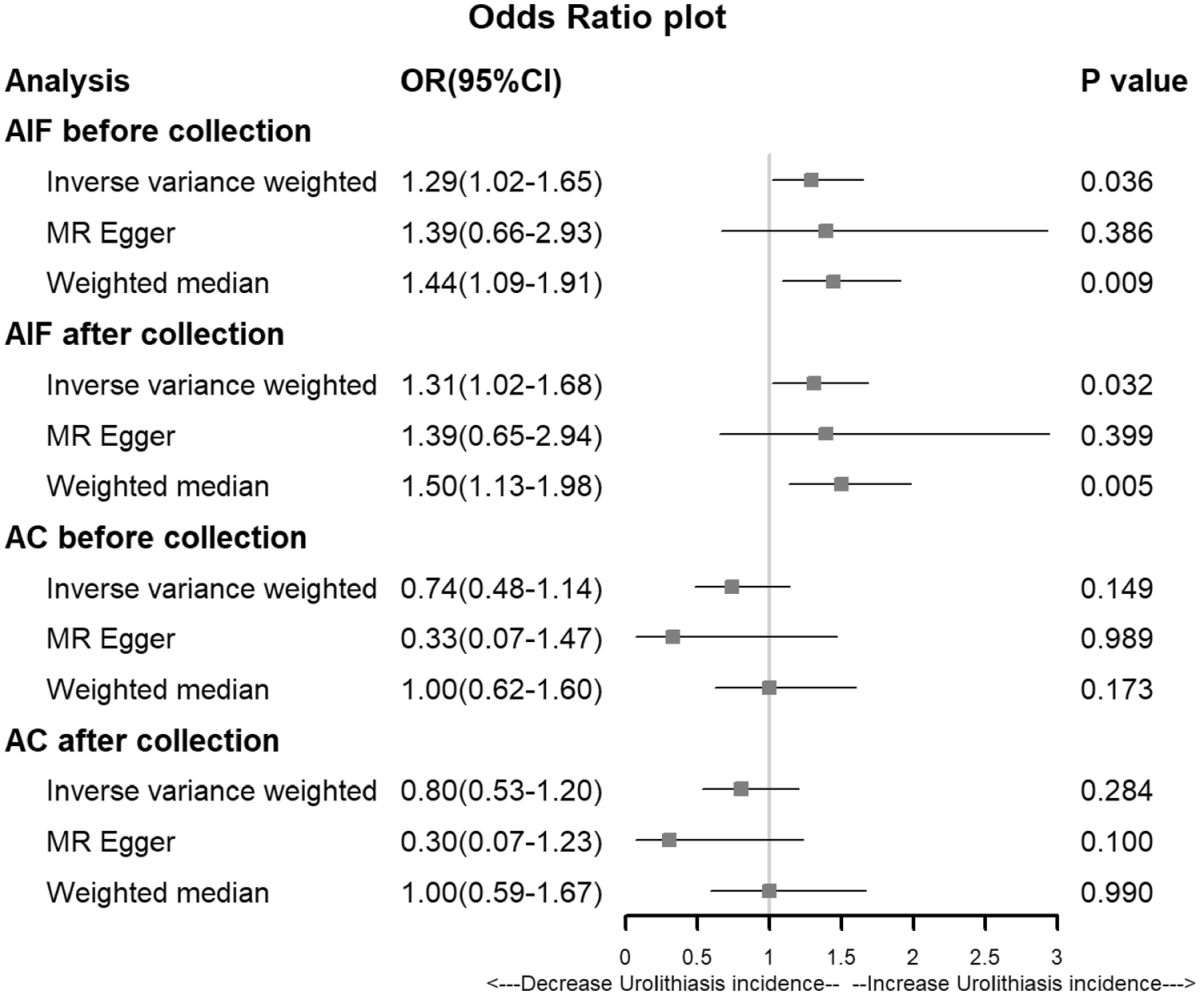
Odds ratio plot for alcohol and urolithiasis. *AIF* alcohol intake frequency; *AC* alcohol consumption; *OR* odds ratio

The results of the MR analysis revealed a borderline significant causal relationship between AIF and the risk of urolithiasis. This was determined by the IVW (OR (95% CI) = 1.29 (1.02, 1.65), *p* = 0.036) and weighted median (OR (95% CI) = 1.44 (1.10, 1.89), *p* = 0.008) approaches. The MR-Egger model provided similar risk estimates (OR (95% CI) = 1.39 (0.66, 2.93), *p* = 0.386), but the link was not statistically significant. Heterogeneity was detected by the Cochran *Q*-test, with a *p*-value of 1.12 × 10^–5^ for MR-Egger and 8.63 × 10^–6^ for IVW. The MR-PRESSO also indicated the presence of heterogeneity (*p*-value in the global heterogeneity test < 0.001). After excluding two outliers (rs13178443 and rs4916723), the MR techniques were reapplied. The results showed that AIF significantly increased the risk of urolithiasis according to the IVW approach (OR (95% CI) = 1.31(1.02, 1.68), *p* = 0.032). The MR estimates also became statistically significant, highlighting the strong association between genetically predicted AIF and the likelihood of urolithiasis (Fig. 2).

Figures S1 and S2 illustrate the MR regression slopes and individual causal estimates for each of the 71 SNPs. No evidence of directional pleiotropy was detected, as indicated by the non-significant intercept (intercept = − 0.001; SE = 0.009. *p* = 0.878). The AIF-related variants were found to be associated with an increased risk of urolithiasis. The results of the leave-one-out sensitivity analysis demonstrated that the exclusion of any single SNP did not significantly affect the overall association between AIF and urolithiasis, as shown in Figure S3. This suggests that the findings of our study were not driven by any single genetic variant. Additionally, the funnel plot was symmetrical, suggesting the absence of pleiotropy (Figure S4).

### Causal effect of alcohol consumption on urolithiasis

Sixty-eight SNPs were found to have a substantial and independent association with alcohol consumption. The results of the IVW approach showed no significant impact of alcohol consumption on the risk of urolithiasis (OR (95% CI) = 0.74 (0.48, 1.14), *p* = 0.173). The Cochran *Q*-test showed a *p*-value of 8.63 × 10^–6^, which was comparable to the results obtained from the MR-PRESSO method (*p*-value in the global heterogeneity test < 0.001). After excluding two outliers (rs1421085 and rs7841320), the MR techniques were reapplied to evaluate the relationship between alcohol consumption and urolithiasis risk. Again, the IVW approach indicated no causal effect of alcohol consumption on urolithiasis risk (OR (95% CI) = 0.8 (0.53, 1.20), *p* = 0.284).

Figures S5 and S6 illustrate the MR regression slopes and individual causal estimates for each of the 66 SNPs. No significant intercept was detected (intercept =  −  0.013; SE = 0.009; *p* = 0.16), indicating the absence of directional pleiotropy. Indeed, no apparent causal relationship was observed between AC and urolithiasis. The leave-one-out sensitivity analysis revealed that no single SNP notably challenged the overall impact of AIF on urolithiasis (Figure S7). Additionally, the symmetry of the funnel plot (Figure S8) indicates a lack of pleiotropy.

### Power calculation

With regard to statistical power, we used the mRnd website [24] for the calculation of power, and for the frequency of drinking the power was 0.45 when calculated using the OR value of IVW, and the power was 0.84 when calculated using the OR value of WM, and for the amount of drinking the power was calculated using the OR value of IVW OR value for the calculation, the calculated power was 0.19.

## Discussion

In the first-ever Mendelian randomization study investigating the association between alcohol and urolithiasis, we employed complementary two-sample MR methods to examine the relationship between alcohol intake frequency, alcohol consumption, and urolithiasis, utilizing vast summary-level GWAS data. The results of the study unveiled a causal effect between alcohol intake frequency and urolithiasis, but not alcohol consumption. In other words, a higher frequency of alcohol intake is associated with an increased risk of urolithiasis. The FINNGEN database provided the GWAS summary information for urolithiasis, while the MR-base database furnished the basic genetic instruments, and another set of genetic instruments was derived from a recent GWAS based on DPW, obtained from the Data Repository for the University of Minnesota. Given that the incidence of urolithiasis is intrinsically tied to ethnic differences, the various gene banks have an impact on the research outcomes, and our study holds greater persuasiveness for people of European ancestry.

Previous studies have yielded inconsistent results on the relationship between alcohol and urolithiasis, with some indicating no significant association [11, 25, 26] and others reporting a negative correlation [7, 27–29]. More recently, two large cohort studies conducted in China [6] and Korea [5] have also suggested a negative correlation between alcohol consumption and kidney stone risk. The disparities in these findings may be due to residual confounding factors, such as recall bias and traits that are correlated with alcohol intake. Notably, MR can utilize genetic variants that are reliably associated with a potentially modifiable risk factor to determine its causal role in disease risk, so it is less prone to biases brought on by confounding and measurement errors.

Some researchers point out that the diuretic effect of alcohol may be the potential mechanism of the protective effect of urolithiasis [27]. Alcohol has been suggested to dilute metabolites in the blood and urine [8, 30], inhibit vasopressin secretion, and thereby prevent stone formation [31]. However, different types of drinking have a great influence on the results. For example, the protective effect of red wine may come from its antioxidant effect [32], while beer is its diuretic effect [27]. It is worth noting that alcohol may facilitate the excretion of urinary calcium by decreasing the renal tubular reabsorption of calcium, which could lead to transient hypercalciuria and thus elevate the risk of urolithiasis development [33]. Animal studies have also revealed that rats treated with ethanol develop crystal formation [34]. Additionally, alcohol consumption has been associated with an increased risk of stone formation due to its potential to stimulate the production of uric acid metabolites [35, 36] and induce oxidative stress damage to kidney tissue [9].

In addition, although our Mendelian randomization study found no evidence of a causal relationship between alcohol consumption and urolithiasis risk, excessive alcohol consumption can still be detrimental due to the potentially harmful effects of ethanol metabolites such as acetaldehyde [37]. Alcohol consumption is associated with various adverse health outcomes and can have a significant impact on health across the lifespan, especially in men [3]. Overall, the relationship between alcohol and urolithiasis is multifaceted and not yet fully elucidated. Our findings emphasize the enormous potential of MR in urolithiasis research, providing insights into mechanisms and informing interventions aimed at reducing the incidence and preventing recurrence.

This study has several strengths, including the use of large datasets to examine the relationship between alcohol and urolithiasis, as well as the utilization of an MR study design and multiple single nucleotide polymorphisms (SNPs) as instrumental variables for alcohol. The MR approach helps to minimize bias resulting from reverse causality and confounding factors. Moreover, the study sample primarily consisted of individuals of European ancestry, providing a homogeneous sample for analysis. But current research shows that Finnish people are an isolated and relatively genetically similar population presenting variations in DNA that might predispose them to some metabolic disease [38], so we also carried out some statistical tests. Although DNA in the Finnish population may be associated with certain metabolic diseases, for the present study, the horizontal pleiotropy test in this study was not statistically significant, suggesting that there are no other confounding factors between alcohol consumption and urolithiasis. In the future, it might be possible to further study whether there are metabolic diseases that might be mediators of the two. And the statistical power of alcohol consumption is low, that is, the probability of type-II error is high, which may still need further study. Additionally, limitations of the study also include the method of ascertainment of alcohol data in the MR-base database and GSCAN (GWAS & Sequencing Consortium of Alcohol and Nicotine use) database, which did not distinguish between types of alcohol or relative levels of consumption and population stratification may have impacted the results, and the findings might not apply to non-European populations.

## Conclusion

Our study indicates a likely causal link between alcohol intake frequency and the risk of urolithiasis in individuals of European descent. However, we did not observe evidence of a causal association between alcohol consumption and urolithiasis risk.

### Supplementary Information

Below is the link to the electronic supplementary material.

Supplementary file1 (PDF 2220 KB)

## Data Availability

Data from MR-base (https://gwas.mrcieu.ac.uk/), GSCAN (https://genome.psych.umn.edu/index.php/GSCAN) and FinnGen consortium (https://www.finngen.fi/en) are publicly available.
