# Supplementary material for: Association between alcohol and urolithiasis: a mendelian randomization study
Source: Urolithiasis. 2023 Aug 15;51(1):103. doi: 10.1007/s00240-023-01472-0 (PMC10427707; doi:10.1007/s00240-023-01472-0)
Supplement: Supplementary file 1 — Supplementary file1 (PDF 2220 KB) [file 240_2023_1472_MOESM1_ESM.pdf]

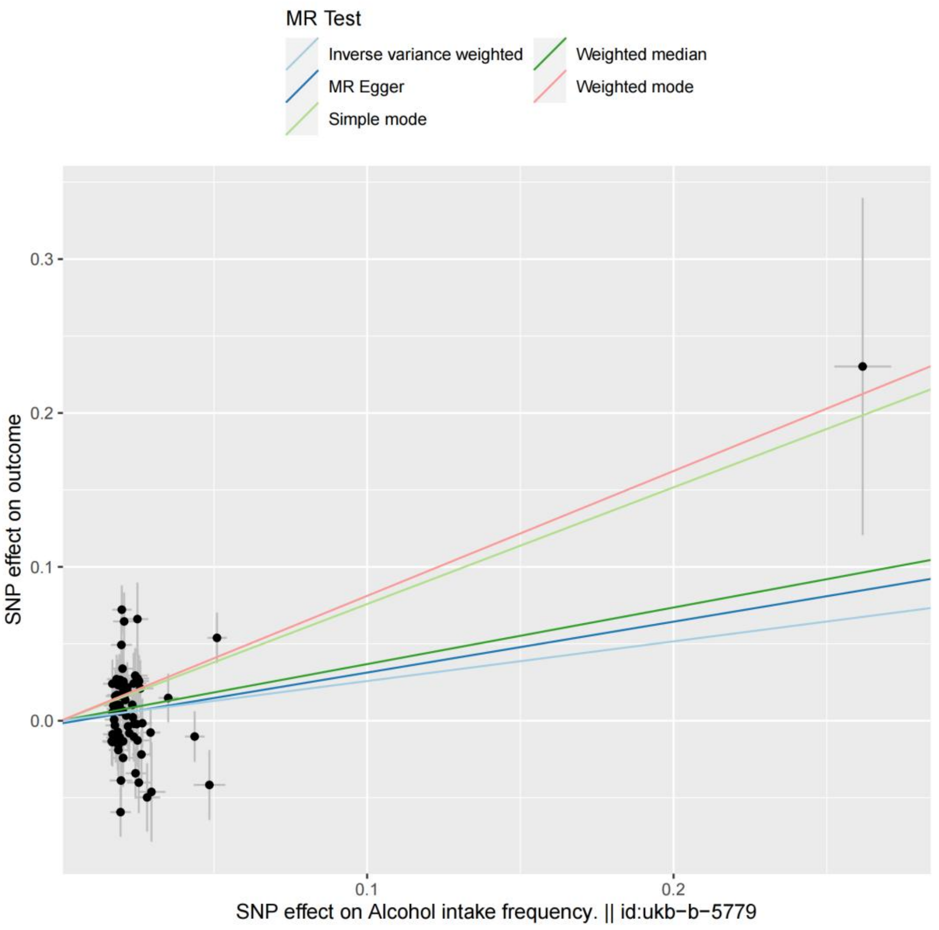

Figure S1. Scatter plot of SNPs associated with AIF and their risk of kidney stone after outliers removal with MR-PRESSO .

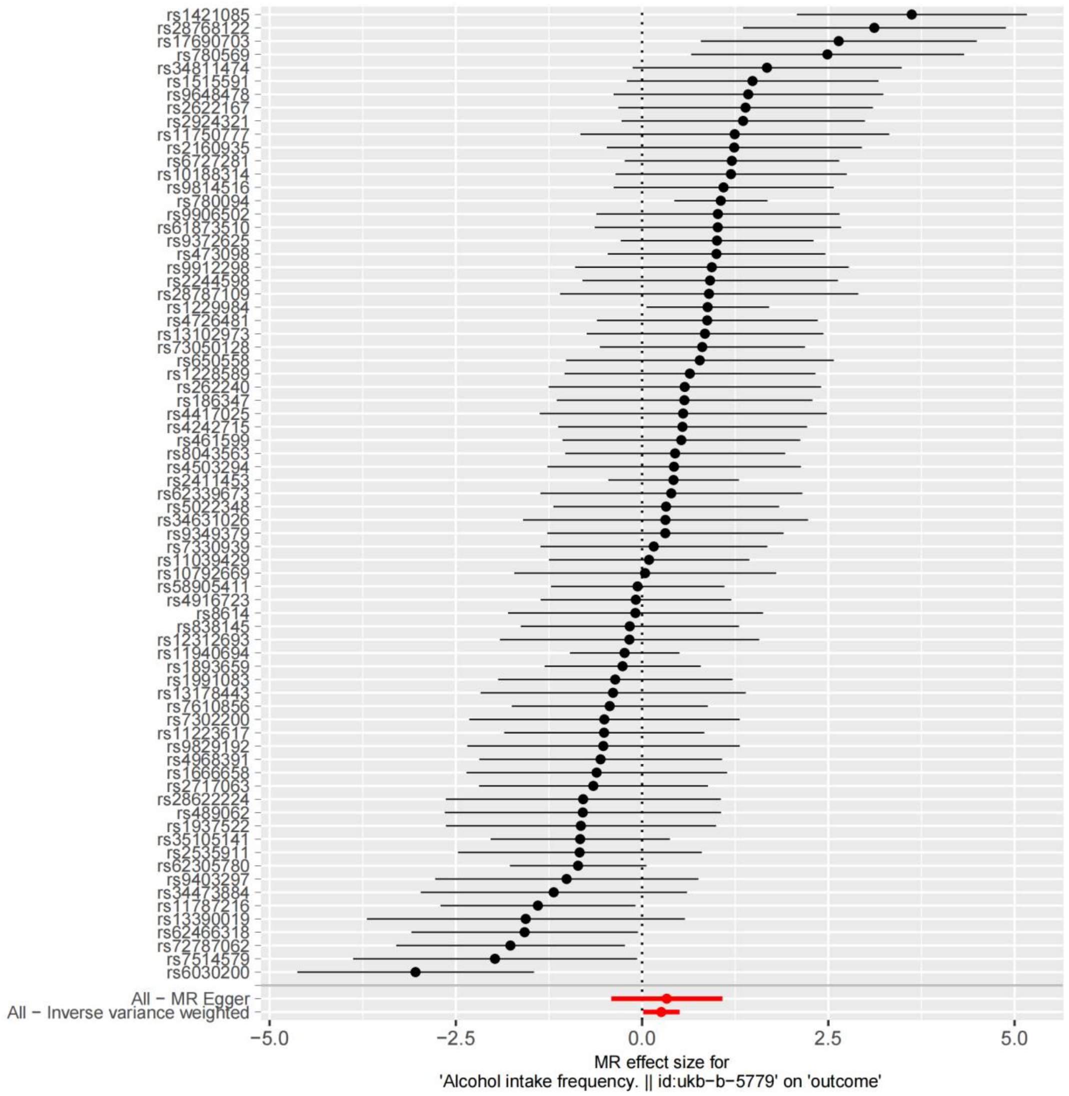

Figure S2. Forest plot of SNPs associated with AIF and their risk of kidney stone after outliers removal with MR-PRESSO .

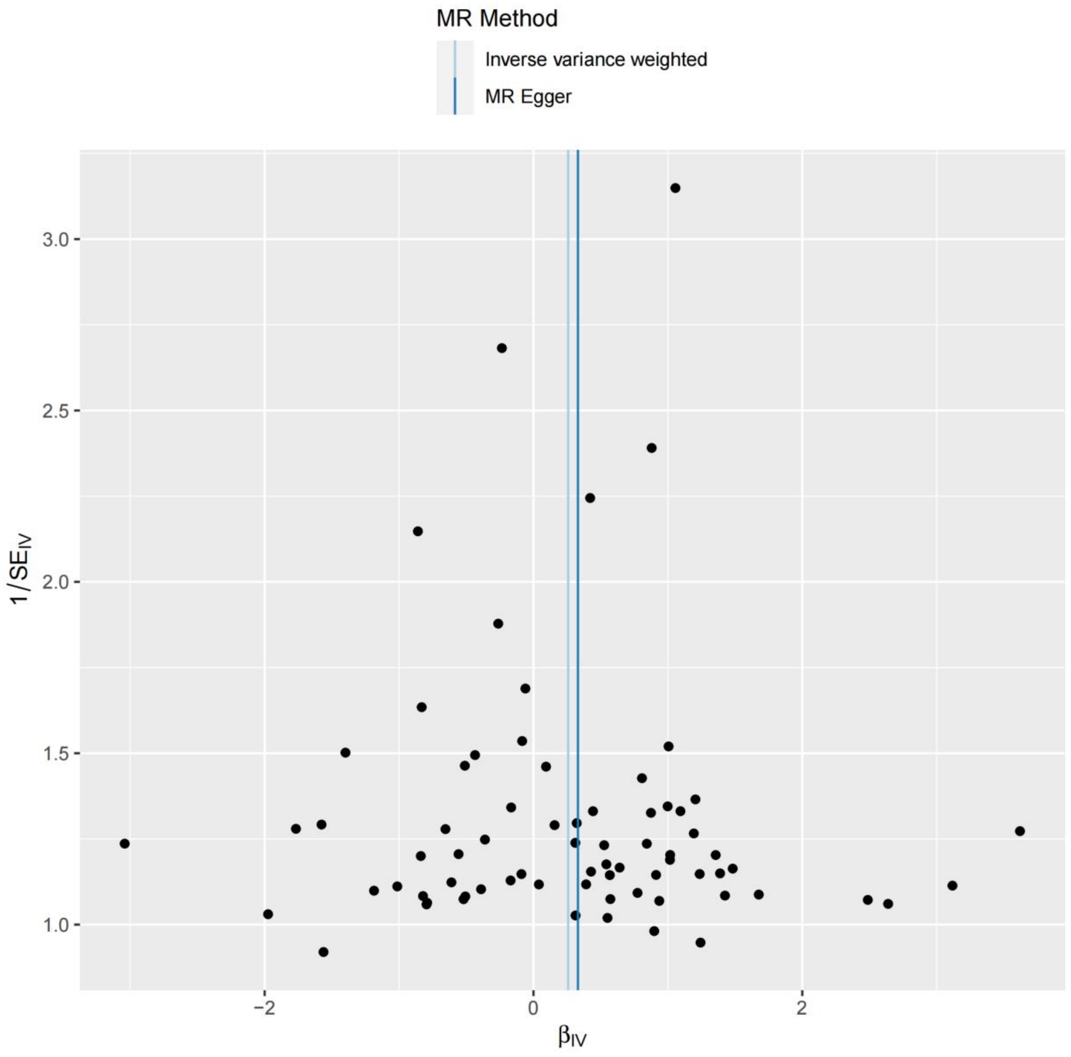

Figure S3. Funnel plot of SNPs associated with AIF and their risk of kidney stone after outliers removal with MR-PRESSO .

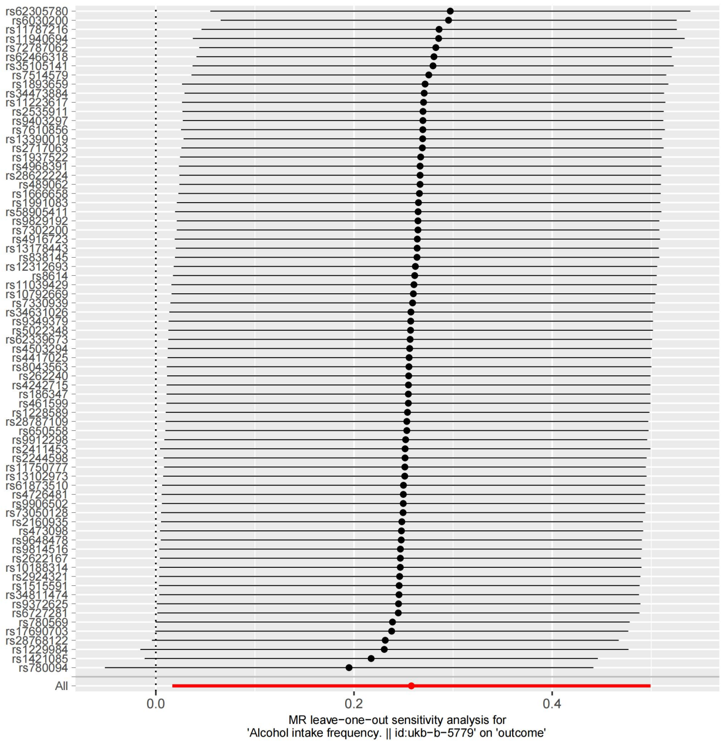

Figure S4. Leave-one-out of SNPs associated with AIF and their risk of kidney stone after outliers removal with MR-PRESSO.

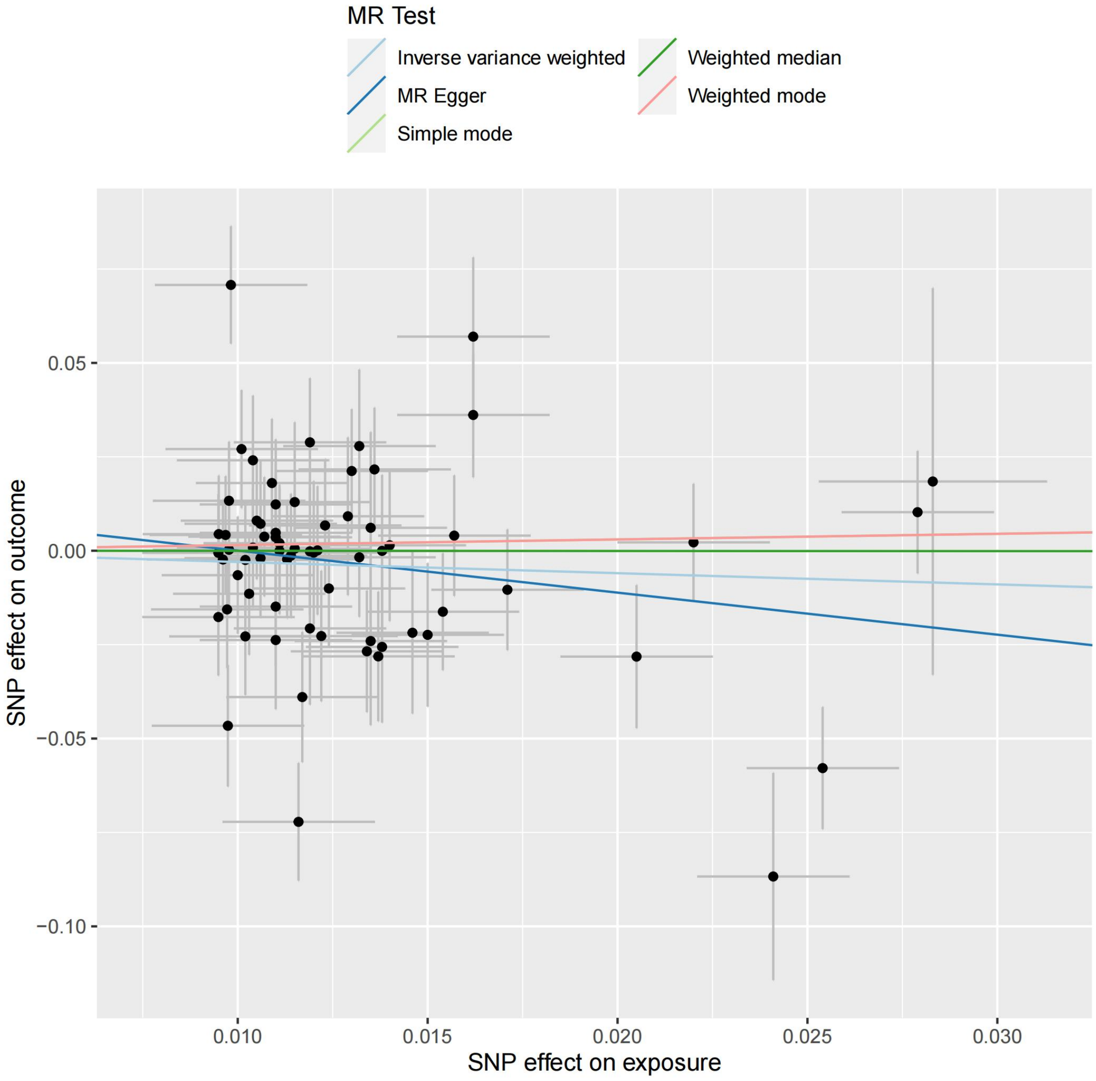

Figure S5. Scatter plot of SNPs associated with AC and their risk of kidney stone after outliers removal with MR-PRESSO .

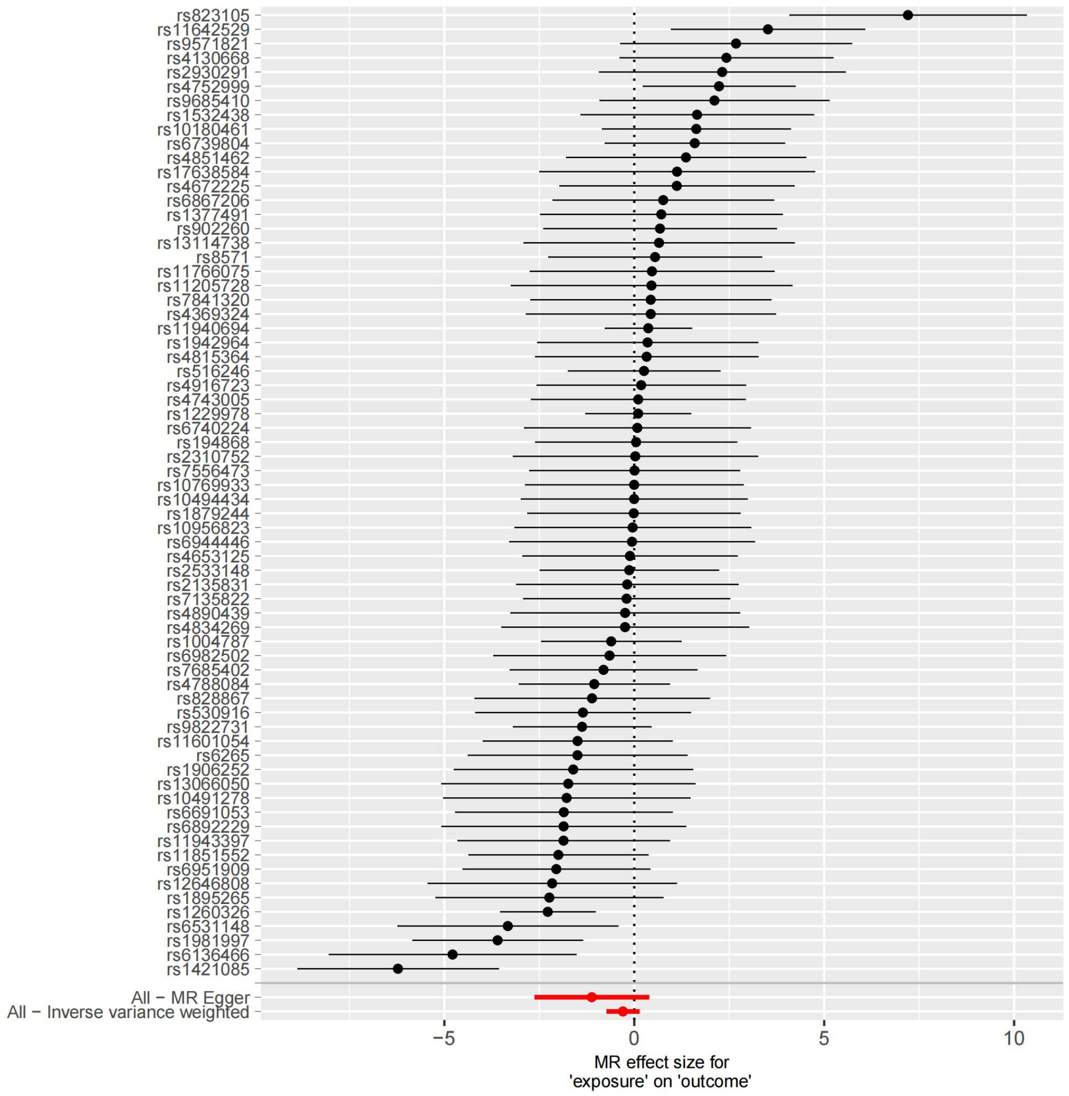

Figure S2. Forest plot of SNPs associated with AC and their risk of kidney stone after outliers removal with MR-PRESSO .

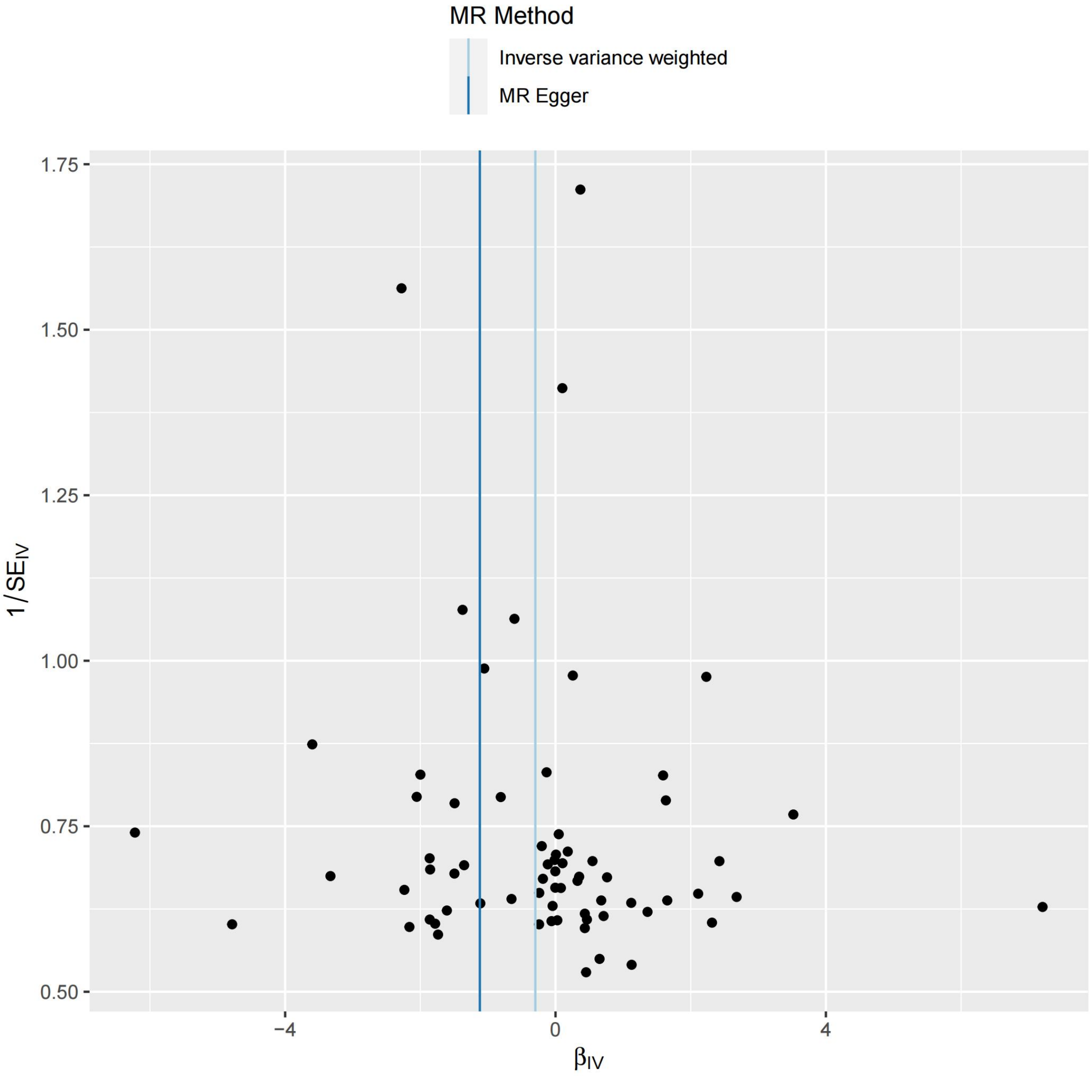

Figure S7. Funnel plot of SNPs associated with AC and their risk of kidney stone after outliers removal with MR-PRESSO .

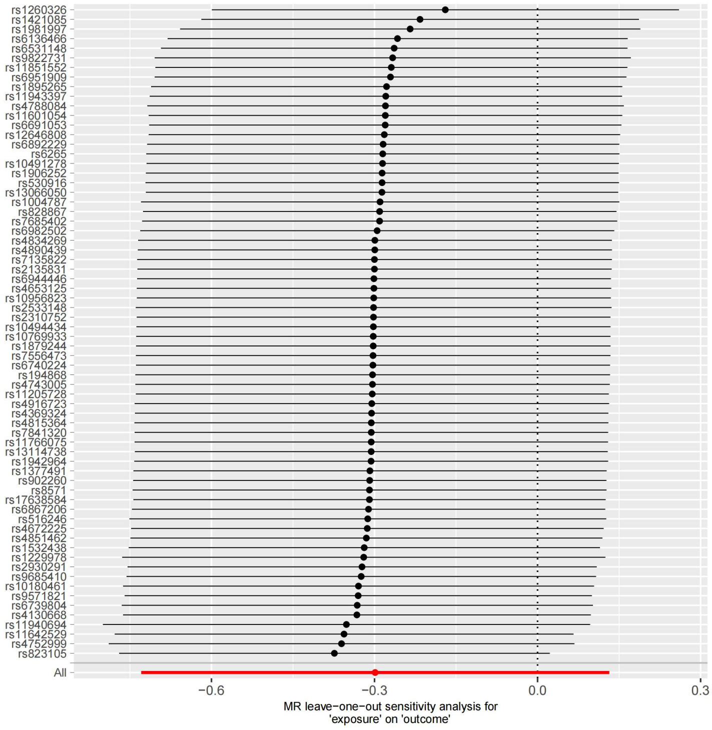

Figure S8. Leave-one-out of SNPs associated with AC and their risk of kidney stone after outliers removal with MR-PRESSO.

Supplementary Table 1. 99 SNPs associated with major AIF

SNP	Chromosome	Position	Effect allele	Other allele	Frequency	Beta	Se	P	samplesize
rs780569	1	4569436	A	T	0.70882	0.0198033	0.00336454	4.00E-09	462346
rs4503294	1	940096	T	C	0.565333	0.0181476	0.00307049	3.40E-09	462346
rs28787109	1	51218695	A	G	0.40423	0.0178107	0.00308461	7.70E-09	462346
rs2244598	1	216681000	C	T	0.605114	-0.018378	0.00311907	3.80E-09	462346
rs4417025	1	35363679	A	G	0.361153	-0.0188379	0.00316516	2.70E-09	462346
rs7514579	1	94051350	C	A	0.232457	0.0196672	0.00359788	4.60E-08	462346
rs2717063	2	58110969	A	C	0.585731	-0.0203704	0.00308457	4.00E-11	462346
rs6727281	2	65558588	T	C	0.184023	-0.024322	0.00391964	5.50E-10	462346
rs780094	2	27741237	C	T	0.615206	-0.0509938	0.00310506	1.30E-60	462346
rs13390019	2	97797680	C	T	0.134041	0.0296116	0.00449182	4.30E-11	462346
rs10188314	2	215402926	T	C	0.470852	-0.0197869	0.00303605	7.20E-11	462346
rs4241258	2	74226102	T	C	0.13763	0.0250636	0.00440325	1.30E-08	462346
rs72769229	2	2220795	T	A	0.154942	-0.0231352	0.00419154	3.40E-08	462346
rs17662759	2	193989223	C	T	0.089115	0.0301348	0.00546031	3.40E-08	462346
rs1991083	2	23887437	T	C	0.679886	-0.0223925	0.00325813	6.30E-12	462346
rs473098	2	45139779	T	C	0.557689	-0.0217406	0.00304345	9.10E-13	462346
rs9829192	3	38569463	T	G	0.435133	0.0169324	0.00305032	2.80E-08	462346
rs76082653	3	49029468	T	C	0.054327	0.0464269	0.00668673	3.80E-12	462346
rs262240	3	68408109	T	C	0.468553	-0.017207	0.00303483	1.40E-08	462346
rs9814516	3	85407980	T	G	0.237423	-0.0251117	0.00355589	1.60E-12	462346
rs7610856	3	71579022	A	C	0.429053	-0.023864	0.00307022	7.70E-15	462346
rs1515591	3	174213976	G	T	0.383186	0.0182303	0.00311635	4.90E-09	462346
rs1228589	3	131634826	A	G	0.246133	0.0210699	0.00352806	2.30E-09	462346
rs28622224	4	55088093	T	C	0.280364	-0.0186203	0.00336834	3.20E-08	462346
rs13135092	4	103198082	G	A	0.083483	0.0438341	0.00549884	1.60E-15	462346
rs11940694	4	39414993	G	A	0.604193	-0.0437138	0.00311609	1.00E-44	462346
rs362307	4	3241845	T	C	0.074582	0.0433047	0.00580219	8.40E-14	462346
rs1229984	4	100239319	C	T	0.97277	-0.261708	0.00918496	1.40E-178	462346
rs13102973	4	135900688	C	T	0.61881	-0.0194072	0.00311875	4.90E-10	462346
rs62339673	4	184828533	A	C	0.626705	0.0182943	0.00315406	6.60E-09	462346
rs34811474	4	25408838	A	G	0.230728	-0.0201809	0.00359305	1.90E-08	462346
rs2159935	4	55521017	A	G	0.490369	-0.0185742	0.00302582	8.30E-10	462346
rs62305780	4	100290815	G	C	0.102253	-0.0485216	0.00506585	9.90E-22	462346
rs13178443	5	145615275	T	C	0.276349	-0.0186516	0.00338983	3.80E-08	462346
rs11750777	5	166830787	A	G	0.209454	-0.020493	0.00372613	3.80E-08	462346
rs4916723	5	87854395	C	A	0.420617	0.0239479	0.00309951	1.10E-14	462346
rs461599	5	144136931	C	A	0.462259	-0.0191888	0.00303977	2.70E-10	462346
rs56194430	5	67824690	T	C	0.16931	0.0225403	0.00407148	3.10E-08	462346

rs9403297	6	141705482	A	G	0.372967	0.0188234	0.00313037	1.80E-09	462346
rs9349379	6	12903957	G	A	0.405493	-0.0193455	0.00308215	3.50E-10	462346
rs12153855	6	32074804	C	T	0.10497	0.0294435	0.00493484	2.40E-09	462346
rs9372625	6	98344031	A	G	0.381706	-0.0255579	0.00312467	2.90E-16	462346
rs62466318	7	73042085	T	C	0.202827	-0.0254919	0.0037742	1.40E-11	462346
rs2622167	7	153486704	A	G	0.428653	-0.0191155	0.0030674	4.60E-10	462346
rs73050128	7	1961882	A	C	0.164488	-0.0260048	0.00409088	2.10E-10	462346
rs6943160	7	99872071	C	T	0.208646	0.020627	0.00372774	3.10E-08	462346
rs4726481	7	141668403	T	G	0.400576	0.0217614	0.00310188	2.30E-12	462346
rs9648478	7	39325802	A	G	0.510245	0.0168603	0.00302891	2.60E-08	462346
rs2160935	8	30840651	T	C	0.604293	-0.018718	0.00309117	1.40E-09	462346
rs34440851	8	87214346	T	C	0.157151	-0.0226831	0.00415058	4.60E-08	462346
rs11787216	8	142615222	T	C	0.369127	0.0244162	0.00320078	2.40E-14	462346
rs2977454	8	141539923	G	C	0.124072	-0.0259222	0.00459853	1.70E-08	462346
rs74679146	9	16287769	C	T	0.074515	-0.0320735	0.00575757	2.50E-08	462346
rs489062	10	99715744	A	G	0.437454	0.0166498	0.00305291	4.90E-08	462346
rs34473884	10	133761285	A	G	0.24819	-0.0203615	0.00350346	6.20E-09	462346
rs61873510	10	102626510	T	G	0.32785	0.0203737	0.00330311	6.90E-10	462346
rs4242715	10	133986135	A	G	0.680585	-0.0186543	0.00324831	9.30E-09	462346
rs10792669	11	82688356	G	A	0.505254	0.0174322	0.00304065	9.90E-09	462346
rs11223617	11	133780757	A	G	0.206155	0.0250908	0.00375381	2.30E-11	462346
rs550942	11	58394154	T	C	0.823865	0.022401	0.00398884	2.00E-08	462346
rs11039429	11	47867059	T	C	0.454624	-0.0235595	0.00303737	8.70E-15	462346
rs1666658	11	121801129	C	T	0.392206	0.0179674	0.00309866	6.70E-09	462346
rs12312693	12	57511734	C	T	0.451772	-0.0176811	0.00305026	6.80E-09	462346
rs7302200	12	56449435	A	G	0.339998	-0.0184222	0.00319841	8.40E-09	462346
rs28768122	12	123885974	C	T	0.759525	0.0207	0.00355204	5.60E-09	462346
rs7298932	12	23727301	G	A	0.147849	-0.0237214	0.00431166	3.80E-08	462346
rs58905411	12	54623132	A	G	0.410052	-0.0266343	0.00307848	5.10E-18	462346
rs1937522	13	68080817	G	A	0.528054	0.0168979	0.00303207	2.50E-08	462346
rs7330939	13	49971400	T	C	0.720352	-0.0213301	0.0034046	3.70E-10	462346
rs2535911	14	73523162	T	C	0.354749	-0.0188476	0.00316849	2.70E-09	462346
rs186347	14	59072226	T	G	0.463343	0.0179489	0.00305071	4.00E-09	462346
rs80292319	15	76508632	C	T	0.057704	-0.0393728	0.00649583	1.40E-09	462346
rs117799466	15	34659517	C	G	0.336989	-0.0196704	0.00331968	3.10E-09	462346
rs34631026	16	6172126	T	C	0.446061	-0.0169128	0.00304832	2.90E-08	462346
rs72787062	16	72105844	A	G	0.162767	-0.0281947	0.00410324	6.40E-12	462346
rs35105141	16	30057148	T	C	0.401541	0.026345	0.00308788	1.40E-17	462346
rs1421085	16	53800954	C	T	0.403447	0.0199392	0.00308481	1.00E-10	462346
rs1104608	16	73912588	C	G	0.426338	0.0174212	0.00308849	1.70E-08	462346
rs8043563	16	19982353	C	G	0.737192	0.0233654	0.00347125	1.70E-11	462346
rs2411453	16	28632021	G	T	0.597353	-0.0350793	0.00309039	7.30E-30	462346
rs728538	16	51205819	G	T	0.168868	0.0228752	0.00406259	1.80E-08	462346
rs9906502	17	7615745	A	G	0.176998	0.0237883	0.00396178	1.90E-09	462346

rs8614	17	27588806	A	C	0.182509	0.0247806	0.00392537	2.70E-10	462346
rs4968391	17	57780943	T	G	0.674892	-0.0192695	0.0032265	2.30E-09	462346
rs9912298	17	29735752	C	A	0.239585	0.0205894	0.00358993	9.70E-09	462346
rs17690703	17	43925297	T	C	0.262687	0.0250342	0.00343021	2.90E-13	462346
rs650558	17	40721042	T	C	0.247918	0.0207362	0.0035079	3.40E-09	462346
rs1893659	18	21080859	A	C	0.459939	-0.029326	0.00305313	7.60E-22	462346
rs5022348	18	22639237	T	C	0.40703	0.0202641	0.00357005	1.40E-08	462346
rs2043677	18	38313195	T	C	0.145599	0.0261133	0.0043272	1.60E-09	462346
rs9958320	18	38269812	C	T	0.153147	0.0248553	0.00427092	5.90E-09	462346
rs62097995	18	50843233	A	T	0.423591	0.0200024	0.00306682	6.90E-11	462346
rs2924321	18	53125435	A	G	0.539592	-0.0195131	0.00305022	1.60E-10	462346
rs4940926	18	57732418	C	T	0.735045	-0.0191003	0.00344068	2.80E-08	462346
rs838145	19	49248730	A	G	0.542982	0.0219548	0.00305549	6.70E-13	462346
rs6030200	20	35554361	A	G	0.31415	-0.019529	0.00327075	2.40E-09	462346
rs11700855	21	34270051	G	A	0.093465	-0.029795	0.00523292	1.20E-08	462346
rs71651683	22	24828853	T	C	0.0142	-0.0704589	0.0127906	3.60E-08	462346
rs1894544	22	48881562	C	G	0.454379	0.0173933	0.00304627	1.10E-08	462346

Supplementary Table 2. 76 SNPs associated with major AC

SNP	Effect allele	Other allele	Frequency	Beta	Se	P	samplesize	F-statistics	select
rs7556473	A	G	0.253	-0.0121	0.002	1.15E-09	665054	13.83533236	T
rs4653125	T	C	0.673	0.0114	0.002	1.88E-09	628236	14.30049357	T
rs11205728	C	T	0.153	-0.0135	0.002	1.92E-08	665054	11.80915405	T
rs2310752	G	A	0.425	-0.00977	0.002	2.57E-08	665054	11.66332069	T
rs10494434	A	G	0.685	-0.0111	0.002	3.11E-09	665054	13.2930446	T
rs6691053	C	T	0.208	-0.0138	0.002	1.24E-10	665054	15.68648473	T
rs823105	G	A	0.443	0.00982	0.002	1.82E-08	665054	11.89757263	T
rs6531148	T	C	0.758	-0.0117	0.002	7.58E-09	666978	12.55547572	T
rs1260326	T	C	0.615	0.0254	0.002	3.16E-46	666978	76.38739796	T
rs1004787	G	A	0.551	0.0171	0.002	1.13E-22	666978	36.17282403	T
rs4672225	C	T	0.276	-0.011	0.002	1.26E-08	666978	12.08953488	T
rs6739804	T	C	0.684	-0.0136	0.002	2.85E-13	666978	19.98953626	T
rs828867	G	A	0.551	0.0103	0.002	3.44E-09	666978	13.12349882	T
rs4851462	T	C	0.384	0.00977	0.002	4.14E-08	666978	11.28956263	T
rs10180461	T	C	0.382	-0.013	0.002	3.21E-13	666978	19.94895883	T
rs6740224	A	C	0.307	-0.0104	0.002	3.37E-08	666978	11.50573806	T
rs1377491	A	T	0.798	0.0129	0.002	2.20E-09	666978	13.41254268	T
rs13066050	C	T	0.207	0.0119	0.002	2.50E-08	666978	11.62287924	T
rs9822731	T	C	0.227	0.0205	0.002	2.60E-23	666978	36.87277166	T
rs1714507	T	A	0.553	-0.0101	0.002	6.75E-09	666978	12.60817748	T
rs1879244	C	T	0.738	0.0119	0.002	1.32E-09	666978	13.69081155	T
rs12646808	T	C	0.333	-0.011	0.002	2.47E-09	666978	13.43794594	T
rs11940694	A	G	0.608	0.0279	0.002	9.51E-56	666978	92.77418752	T
rs16854020	G	A	0.124	0.0189	0.003	6.28E-13	666978	8.622658737	F
rs7685402	G	T	0.468	0.0124	0.002	9.85E-13	666978	19.14176682	T
rs1229978	T	C	0.43	0.022	0.002	1.93E-36	666978	59.31929739	T
rs13114738	C	T	0.072	-0.0283	0.003	2.92E-17	666978	11.89179108	T
rs4834269	T	C	0.425	0.00961	0.002	4.07E-08	666978	11.2844293	T
rs11943397	T	C	0.634	0.0122	0.002	1.23E-11	666978	17.26910981	T
rs9685410	T	A	0.816	-0.0132	0.002	4.00E-09	666978	13.08076259	T
rs6867206	T	C	0.429	-0.0105	0.002	1.93E-09	666978	13.50359777	T
rs4916723	A	C	0.423	-0.0111	0.002	2.74E-10	666978	15.03628784	T
rs10491278	A	C	0.166	-0.0135	0.002	2.96E-08	611077	12.61592867	T
rs6892229	T	G	0.545	-0.00949	0.002	4.86E-08	666978	11.16648011	T
rs17638584	G	T	0.23	0.0115	0.002	2.10E-08	666978	11.710908	T
rs1895265	A	C	0.516	0.0102	0.002	3.42E-09	666978	12.99189698	T
rs1906252	C	A	0.482	0.00972	0.002	2.05E-08	666978	11.7946677	T
rs11766075	T	C	0.447	0.0095	0.002	4.87E-08	666978	11.15464698	T

rs6951909	C	T	0.264	-0.0137	0.002	3.01E-12	666978	18.23490272	T
rs10155966	A	G	0.127	-0.0158	0.003	1.18E-09	666978	6.150668596	F
rs194868	T	C	0.47	0.0115	0.002	3.66E-11	666978	16.4720949	T
rs6944446	G	A	0.53	0.00949	0.002	4.47E-08	666978	11.21714047	T
rs2533148	T	C	0.485	0.0132	0.002	3.00E-14	666978	21.76104271	T
rs7841320	G	A	0.482	0.00968	0.002	2.31E-08	666978	11.69779029	T
rs10956823	G	T	0.776	0.012	0.002	8.38E-09	666978	12.51552532	T
rs6982502	C	T	0.527	0.01	0.002	7.07E-09	666978	12.46374553	T
rs4743005	G	A	0.178	-0.014	0.002	5.57E-10	666978	14.33923327	T
rs2789514	G	A	0.879	0.0145	0.003	4.13E-08	666978	4.969350957	F
rs4369324	G	T	0.254	-0.011	0.002	3.25E-08	666978	11.46394466	T
rs10769933	G	C	0.82	-0.0138	0.002	8.71E-10	666978	14.05472602	T
rs6265	C	T	0.183	-0.0146	0.002	6.63E-11	666978	15.93521531	T
rs4752999	C	T	0.325	-0.0162	0.002	1.63E-18	666978	28.78754364	T
rs7118897	G	A	0.26	-0.0114	0.002	4.23E-08	602156	12.50237005	T
rs11601054	G	A	0.358	-0.015	0.002	7.76E-17	666978	25.85747488	T
rs11605078	C	T	0.124	0.0144	0.003	3.84E-08	666978	5.005416474	F
rs530916	A	G	0.565	0.011	0.002	4.22E-10	650123	14.86968185	T
rs2135831	A	G	0.542	-0.0106	0.002	1.18E-09	666978	13.94614826	T
rs7135822	G	A	0.479	-0.0113	0.002	8.04E-11	666978	15.9334272	T
rs902260	G	T	0.617	-0.0106	0.002	2.62E-09	666978	13.27617643	T
rs9571821	G	T	0.588	-0.0101	0.002	9.74E-09	666978	12.35645914	T
rs11851552	C	T	0.714	-0.0134	0.002	2.71E-12	666978	18.33388409	T
rs11638215	A	C	0.643	-0.0108	0.002	1.94E-09	666977	13.38764289	T
rs2930291	G	A	0.357	-0.0104	0.002	7.90E-09	666977	12.41431192	T
rs1532438	C	G	0.33	0.0109	0.002	3.65E-09	666977	13.13466477	T
rs17177078	C	T	0.06	-0.0263	0.004	4.96E-13	665054	4.876435591	F
rs4788084	C	T	0.421	-0.0154	0.002	2.08E-18	665054	28.90611163	T
rs1421085	T	C	0.41	-0.0116	0.002	8.00E-11	648199	16.27539043	T
rs11642529	G	C	0.145	0.0162	0.002	5.07E-11	665054	16.26834852	T
rs8571	G	A	0.302	0.0123	0.002	6.52E-11	665054	15.9459978	T
rs4130668	T	C	0.775	0.0119	0.002	1.77E-08	645018	12.34681993	T
rs1981997	G	A	0.205	-0.0241	0.002	2.42E-29	666978	47.33197163	T
rs4890439	G	A	0.386	0.0102	0.002	1.06E-08	666978	12.32913901	T
rs1942964	T	G	0.494	-0.0107	0.002	3.89E-09	611077	14.30947742	T
rs516246	C	T	0.487	0.0157	0.002	1.74E-19	665346	30.791754	T
rs6136466	C	T	0.402	-0.00974	0.002	3.50E-08	666978	11.40305654	T
rs4815364	G	A	0.618	0.011	0.002	5.75E-10	666978	14.28286102	T
